# Surgical exposure to posterolateral quadrant tibial plateau fractures: an anatomic comparison of posterolateral and posteromedial approaches

**DOI:** 10.1186/s13018-022-03236-9

**Published:** 2022-07-15

**Authors:** Sunjun Hu, Shijie Li, Shimin Chang, Shouchao Du, Wenfeng Xiong

**Affiliations:** Department of Orthopedic Surgery, Yangpu Hospital, Tongji University School of Medicine, 450 Tengyue Road, Shanghai, 200090 People’s Republic of China

**Keywords:** Tibial plateau, Posterolateral quadrant, Plateau fracture, Posterior approach, Anterior tibial artery, Posterolateral corner, Plate fixation

## Abstract

**Background:**

Management of posterolateral tibial plateau quadrant fractures can be challenging, and two posterior approaches were frequently used for exposure, reduction, and fixation: posterolateral approach and posteromedial approach. The purpose of this study was to compare their deep anatomical structure and analyze their limits and the risk of injury to important structures during surgical dissection of two approaches.

**Method:**

Five lower limb specimens were used in this study. After dissection of the skin and superficial fascia of each specimen, deep structures were dissected via posteromedial and posterolateral approach, and several parameters including perpendicular distance from the anterior tibial artery coursing through the interosseous membrane fissure to the lateral joint line and apex of fibular head and so on were measured and analyzed.

**Result:**

The perpendicular distance from the ATA coursing through the interosseous membrane fissure to the lateral joint line was 49.3 ± 5.6 mm (range 41.3–56.7 mm), while the distance to the apex of fibular head was 37.7 ± 7.2 mm (range 29.0–48.0 mm). The transverse distance of the anterior tibial vascular bundle is around 10 mm. The perpendicular distance from the top accompanying vein of the ATA bundle to lateral joint line and apex of fibular head was 44.1 ± 6.3 mm and 32.5 ± 7.6 mm, respectively. The maximum proportion of posterolateral tibial plateau shielded by the fibular head from the posterior view was 61.7 ± 4.9% (range 55.6–64.1%). The average length of popliteus muscle outside the joint was 83.1 ± 6.0 mm (range 76.5–92.2 mm), and the width in the middle was 28.1 ± 4.3 mm (range 26.6–29.1 mm).

**Conclusion:**

Although posterolateral approach seems more direct for exposure of posterolateral quadrant tibial plateau fracture, it has three major disadvantages in deep dissection. Posteromedial approach through the medial board of medial head of gastrocnemius–soleus may be safer, but it was hard for direct visualization of articular surface which limits it usage for only a few cases.

## Background

Along with the extensive application of computed tomography (CT) scan imaging in traumatic orthopedics, posterolateral (PL) tibial plateau fracture has gradually gained clinical attention in recent years [1–3]. Hidden by the fibula head, the fibular collateral ligament (FCL), and posterolateral corner (PLC) structures, in addition to these various artery branches and nerves in posterior side of knee joint, the PL tibial plateau fragment is usually hard for exposure, reduction, and fixation [2]. There are three approach categories clinically used: posterocentral approach (through medial and lateral head of gastrocnemius muscle in popliteal fossa, demanding anatomical dissection of popliteal artery bunches, which is seldom used), posteromedial approach (through the medial board of medial head of gastrocnemius muscle and soleus) [4], and posterolateral approach (through the lateral board of lateral head of gastrocnemius muscle and soleus) which was advocated by several authors [5–9]. There are both advantages and disadvantages for exposure of posterolateral quadrant of tibial plateau through posteromedial and posterolateral approaches, and many studies are available for both approaches [10, 11].

This study compares the deep anatomical structures needed to be dissected for posteromedial and posterolateral approaches by cadaver specimens and discusses the limitations of each approach and the risk for injury of important structures.

## Materials and methods

Five fresh lower extremity adult cadaver specimens (provided by Department of anatomy, Tongji University School of Medicine) were surgically dissected, including three male cases and two female cases, aging 48.2 ± 11.5 years old (range 28–67 years old). None of the specimens had signs of previous injury, abnormality, or disease.

**Fig. 1:**
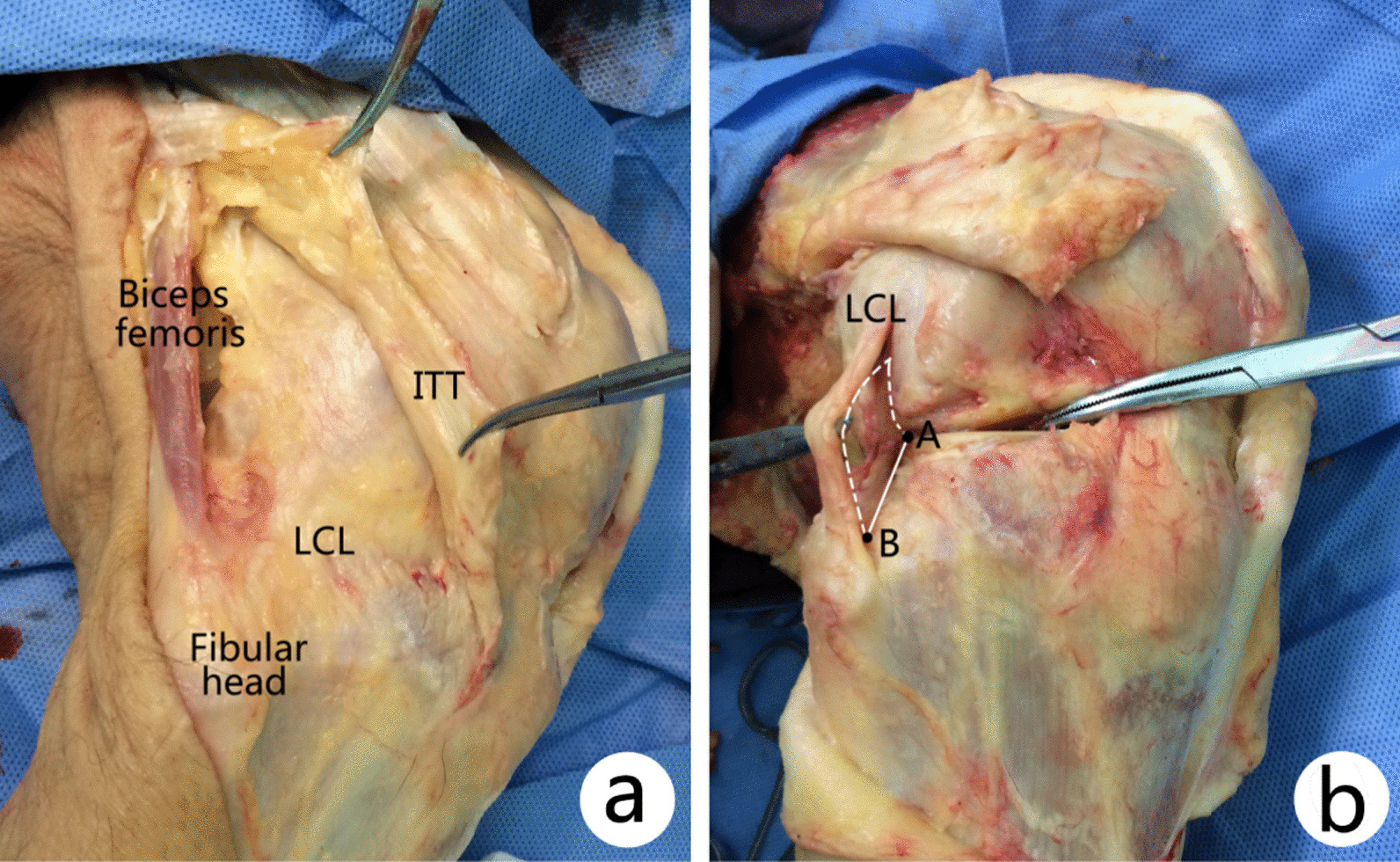
**a** The superficial layer including skin and subcutaneous structure of each specimen was removed, deep structure including LCL and ITT. **b** The distance from the apex of fibular head to the lateral condylar surface rim (AB) was more than 10 mm, which can be used for placing small plate through a modified anterolateral approach. *LCL* lateral collateral ligament or fibular collateral ligament, *ITT* iliotibial tract

Firstly, after dissection of the skin and superficial fascia of each specimen, deep structures were dissected via posteromedial and posterolateral approach. The common peroneal nerve (CPN) was identified on the posterior border of biceps femoris and went down across the popliteal fossa, bypassing the fibular neck. The fibular collateral ligament (FCL) was identified from its origin on the lateral epicondyle of the femur to the head of the fibula below. It does not fuse with either the capsular ligament or the lateral meniscus, and space between the FCL and lateral board of lateral condylar can be used for visualization of fracture or position of small plate through a modified anterolateral approach (Fig. 1).

Secondly, measurement was performed with vernier caliper (correct to 0.1 mm) and tape measure (correct to 1 mm). The following parameters were measured through backside: (1) Perpendicular measurement from the anterior tibial artery coursing through the interosseous membrane fissure to the lateral joint line and apex of fibular head. (2) Perpendicular measurement from the top accompanying vein of the anterior tibial vascular bundle (accompanied by one artery in the middle and one vein each in superior and inferior side) to lateral joint line and apex of fibular head. (3) The percentage of posterolateral tibial plateau shielded by the fibular head from the posterior view. (4) The mean length of popliteus muscle outside the joint and the width in the middle part of popliteus muscle.

## Results

**Fig. 2:**
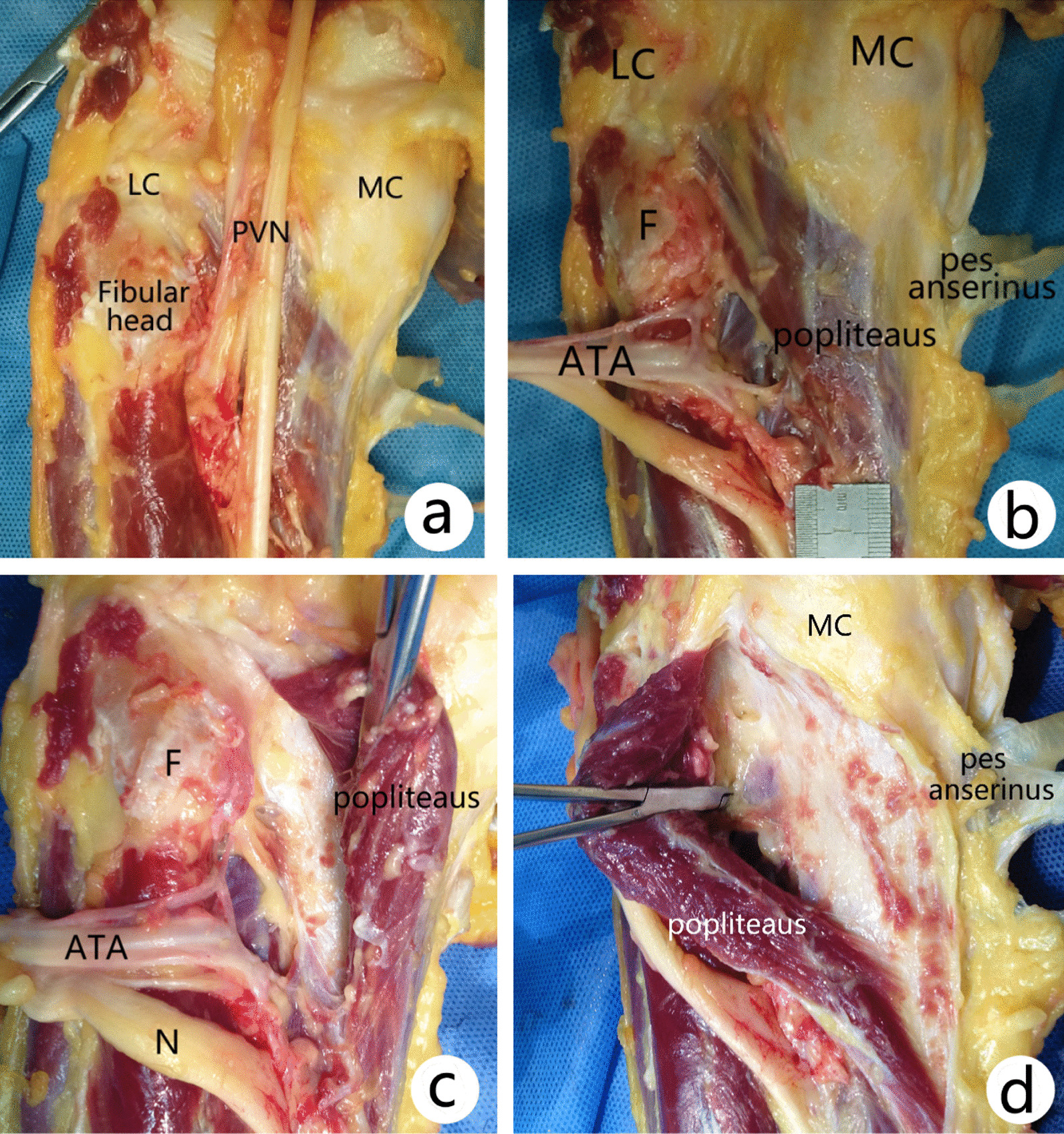
Deep anatomical structures of posterior tibial plateau: The popliteus muscle originates from the lateral surface of the lateral condyle of the femur, passing downward and medially, inserting to the posterior surface of tibia above the soleal line, as a triangle bunchy platymyarian. **a**, **b** Deep structures after superficial layer structures removed. **c** The PL fracture was exposed through a PL approach. **d** The PL fracture was exposed through a PM approach. *LC* lateral condyle, *MC* medial condyle, *PVN* popliteal vascular nerve bundle, *ATA* anterior tibial artery, *F* fibular head

The average height of the five anatomical specimens was 172 ± 4.6 cm (167–178 cm). The popliteal artery courses into anterior tibial artery (ATA) and posterior tibial artery in a bifurcated form at the level of fibular neck. The ATA coursed through the interosseous membrane fissure of superior tibiofibular joint and descended in front of calf. The perpendicular distance from the ATA coursing through the interosseous membrane fissure to the lateral joint line was 49.3 ± 5.6 mm (range 41.3–56.7 mm), while the distance to the apex of fibular head was 37.7 ± 7.2 mm (range 29.0–48.0 mm). The ATA was fixed by interosseous membrane fibers when traversing to anterior space of calf, and excursion was small, so it was easily injured due to distal dissection. The transverse distance of the three vessels of the anterior tibial vascular bundle is around 10 mm. The perpendicular distance from the top accompanying vein of the ATA bundle to lateral joint line and apex of fibular head was (44.1 ± 6.3) mm and (32.5 ± 7.6) mm, respectively, and the shortest distance was 33.9 mm and 21.6 mm, respectively (Fig. 2).

**Fig. 3:**
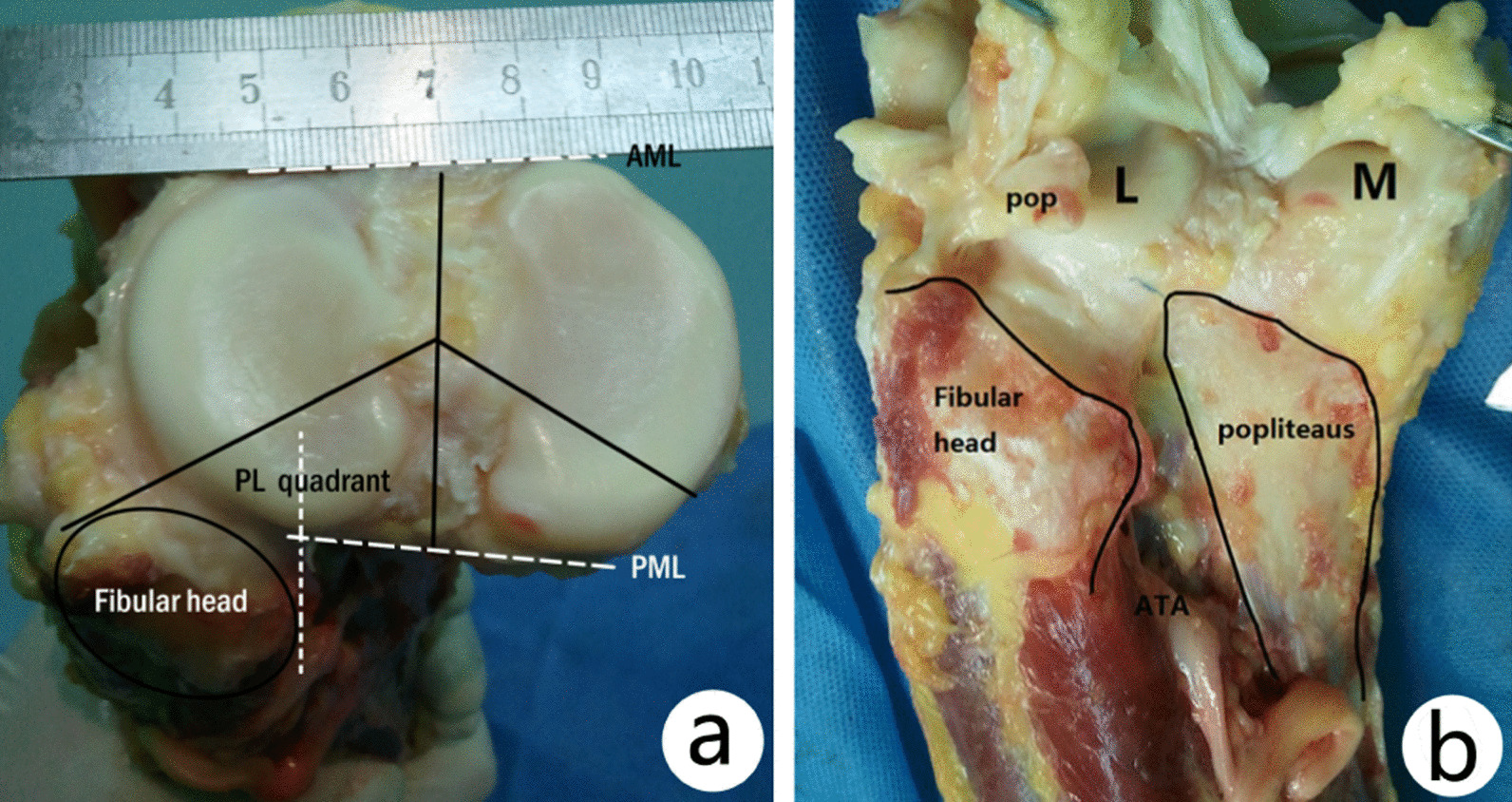
The percentage of posterolateral cortical area shielded by fibular head on transverse plane (**a**) and coronal plane (**b**). On transverse plane, the percentage was more than 50% (**a**). Because of the fibular head, it is difficult to reduce the PL fragment and place the buttress plate via a posterolateral approach. *AML* anterior margin line, *PML* posterior margin line, *POP* popliteal tendon, *L* lateral condyle, *M* medial condyle

The fibular head was a conically inflated structure in proximal end of fibula with the tuberosity structure on lateral surface as the attachment for tendon of biceps femoris and lateral collateral ligament. Joint capsule, posterior, and lateral muscles of calf are also attached to this structure. The maximum proportion of posterolateral tibial plateau shielded by the fibular head from the posterior view was 61.7 ± 4.9% (range 55.6–64.1%) (Fig. 3).

**Fig. 4:**
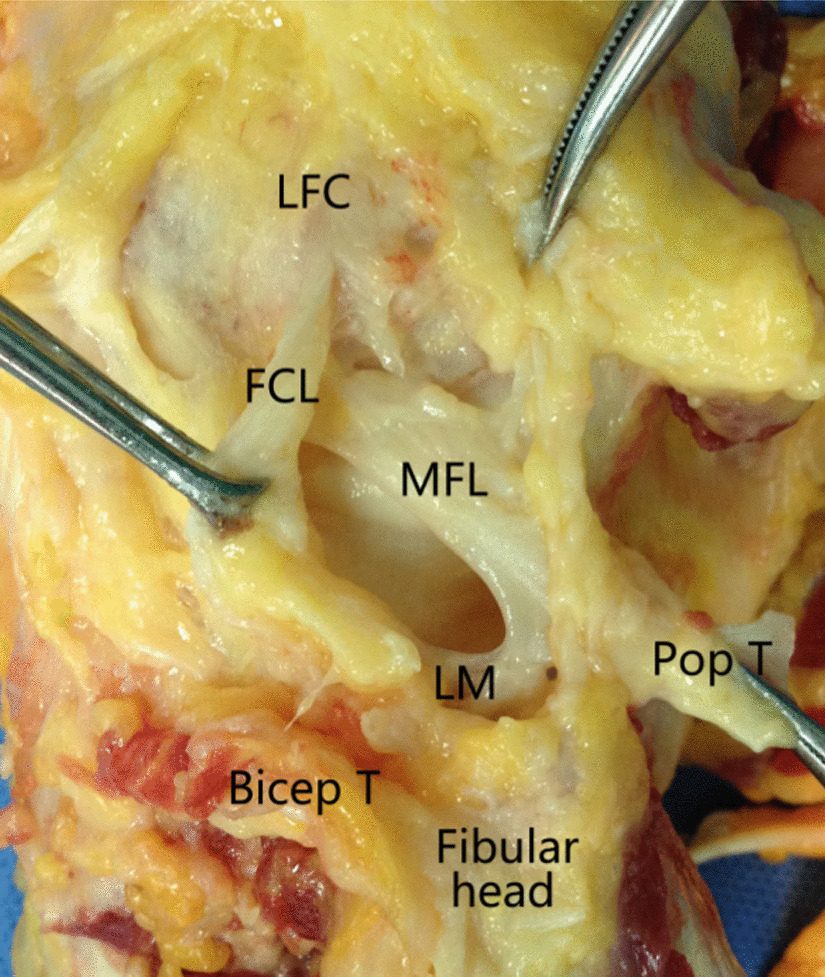
Posterolateral corner structures during dissection via a PL approach. *LFC* lateral femoral condyle, *FCL* fibular collateral ligament, *MFL* posterior meniscofemoral ligament, *LM* lateral meniscus, *Pop T* popliteal tendon

The popliteus muscle originates from the lateral surface of the lateral condyle of the femur as a round tendon, passing downward and medially, inserting to the posterior surface of tibia above the soleal line, as a triangle bunchy platymyarian. The average length of popliteus muscle outside the joint was 83.1 ± 6.0 mm (range 76.5–92.2 mm), and the width in the middle was 28.1 ± 4.3 mm (range 26.6–29.1 mm) (Fig. 2). Except for the popliteus tendon, FCL, biceps femoris tendon, PLC structures including popliteofibular ligament, arcuate ligament, meniscofemoral ligament were also dissected which may be injured during a PL approach (Fig. 4).

## Discussion

The posterolateral (PL) tibial plateau fracture can occur alone or in combination with injuries to anterolateral, posteromedial, or anteromedial quadrants in high-energy fractures, even be associated with anterior cruciate ligament (ACL) incompetence, such as tibial eminence fractures [12, 13]. Most commonly caused by combined eversion and axial force in a flexed or semi-flexed knee position, the isolated PL quadrant tibial plateau fracture is often due to low energy violence such as widely used electric vehicles [12]. According to a morphological study of tibial plateau fractures, 15% of all injuries demonstrated a PL fracture fragment, with an average compression depth of approximately 10 mm [14, 15]. Meulenkamp et al. [2] had reported a prospective study of 65 patients with OTA type B/C tibial plateau fractures comparing outcomes of surgery following submeniscal arthrotomy-assisted reduction or fluoroscopic-guided reduction alone, and postoperative radiological images revealed that locations of most mal-reductions were in the PL quadrants of the tibial plateau. In a cohort of patients with primary anterior cruciate ligament (ACL) tears, Bernholt et al. [16] reported several distinct morphologic variants of lateral tibial plateau impaction fractures, including a pure split, split depression, contained pure depression, and non-contained depression. Giordano et al. [17] suggested a simplified treatment algorithm highlighting two concepts (buttressing and containment) used for plating the PL tibial plateau fragments. Shear-type fractures need buttressing, while non-contained peripheral rim-type fractures need peripheral repair and containment.

There are a sort of surgical excisions used for exposing PL tibial plateau, including conventional AL approach, extended lateral approach, trans-fibular-neck approach, supra-fibular-head approach, and arthroscopy-assisted method [18–20]. As no plate was specially designed for posterolateral fractures, several authors had introduced newly designed plates for fixation, including AL approach and PL approach [21–23]. Giordano et al. [20] proposed a hoop plating technique for the management in cases of extensive posterior tibial plateau articular surface fracture with posterior cortical wall ruptured. Cho et al. [18] had reported using a rim plate in combination with LCP for fixation of the PL fragment via modified anterolateral approach. Chen et al. [21] had designed a rotational support plate and special pressurizer for fixation of the PL fragment directly via the AL approach. However, it is still the mainstream of surgical therapy to expose the fracture through a posterior approach, reduce the fracture, and fix it with a buttress plate.

For a PL approach, lateral sural cutaneous nerve should be protected, which originates from CPN in the popliteal fossa and descends between proper fascia in calf and lateral head of gastrocnemius muscle, while for a PM approach, medial sural cutaneous nerve, great saphenous vein, and saphenous nerve should be carefully protected when superficial dissection.

Several deep structures should be cautioned for a PL approach, which enters from lateral border of lateral head of gastrocnemius muscle. Distal extension of the incision is restricted by the anterior tibial artery (ATA) bunches, so the space after exposing PL tibial plateau was limited, which is generally up to 5 cm in height according to our study. Heidari et al. [24] measured 40 lower extremity samples and found that the ATA coursed through the interosseous membrane at 46.3 ± 9.0 mm (range 27–62 mm) distal to the lateral tibial plateau and 35.7 ± 9.0 mm (range 17–50 mm) distal to the fibula head, which was close to our research. Anatomic variation in the ATA branches may also provide difficulty in dissection of deep structures for both PL and PM approaches. Tindall et al. [25] have reported that 6% of cases originated proximal to the popliteus muscle and passed beneath it in contact with the posterior tibial cortex. The PL fragment was usually shielded by the lateral fibular head and was hardly visualized, and the percentage in our study was up to 61.7%, so it was sometimes forced to cut off partial fibular head for plating. It was also necessary to dissect the popliteus muscle and even cut off its tendon (sutured after the reduction) to expose the PL articular surface, and these manipulations may cause iatrogenic damage to PLC to various degrees. For a PM approach, the dissection used medial board of medial head of gastrocnemius muscle from medial to lateral, exposing and separating partial origin of popliteus muscle. Although this approach may avoid injury to the popliteal neurovascular bundles, it was hard to directly visualize the depressed articular surface fragment, which limits its usage mainly for bicondylar fractures or more complex fractures. It was also difficult to expose posterior proximal tibia cortex and even plating by traction of muscles for a strong patient with bulky gastrocnemius fibers. Huang et al. [26] suggested choosing posteromedial excision during first surgery, for the patients who require to take out the implant after fracture healing. The comparison of the two approaches for an isolated PL fracture is listed in Table 1.

**Table 1:** Surgical exposure to posterolateral quadrant tibial plateau fractures: an anatomic comparison of posterolateral vs posteromedial approaches

	Posterolateral approach	Posteromedial approach
Skin incision	Direct straight incision	Indirect inverted L-shaped incision
Superficial structure	Lateral sural cutaneous nerve	Medial sural cutaneous nerve
		Greater saphenous vein and saphenous nerve
Deep exposure	Via the lateral board of lateral gastrocnemius head and soleus	Via the medial board of medial gastrocnemius head and soleus
Expose of posterolateral tibial plateau	1. Directly, part of the articular surface can be seen	1. Indirect, direct vision of the articular surface is impossible
	2. Superiorly traction of the popliteal muscle or partial dissection	2. Lateral and inferiorly traction of the popliteal muscle
	3. Dissection of the posterolateral corner structure	3. No dissection of the AVN or PLC
	4. Partially shielded by fibular head	
Plate placement	Straightly	Obliquely
Direction of screws	Direct to the middle or medial side	Direct to the lateral side
Dangerous structure	CPN, ATA	Popliteal vessels
Remove the plate	Very hard, may injury the ATA or ATV	Hard for strong persons
Clinical usage	Isolated PL fractures	Bicondylar posterior fractures
	Complex fractures	Complex fractures

**Fig. 5:**
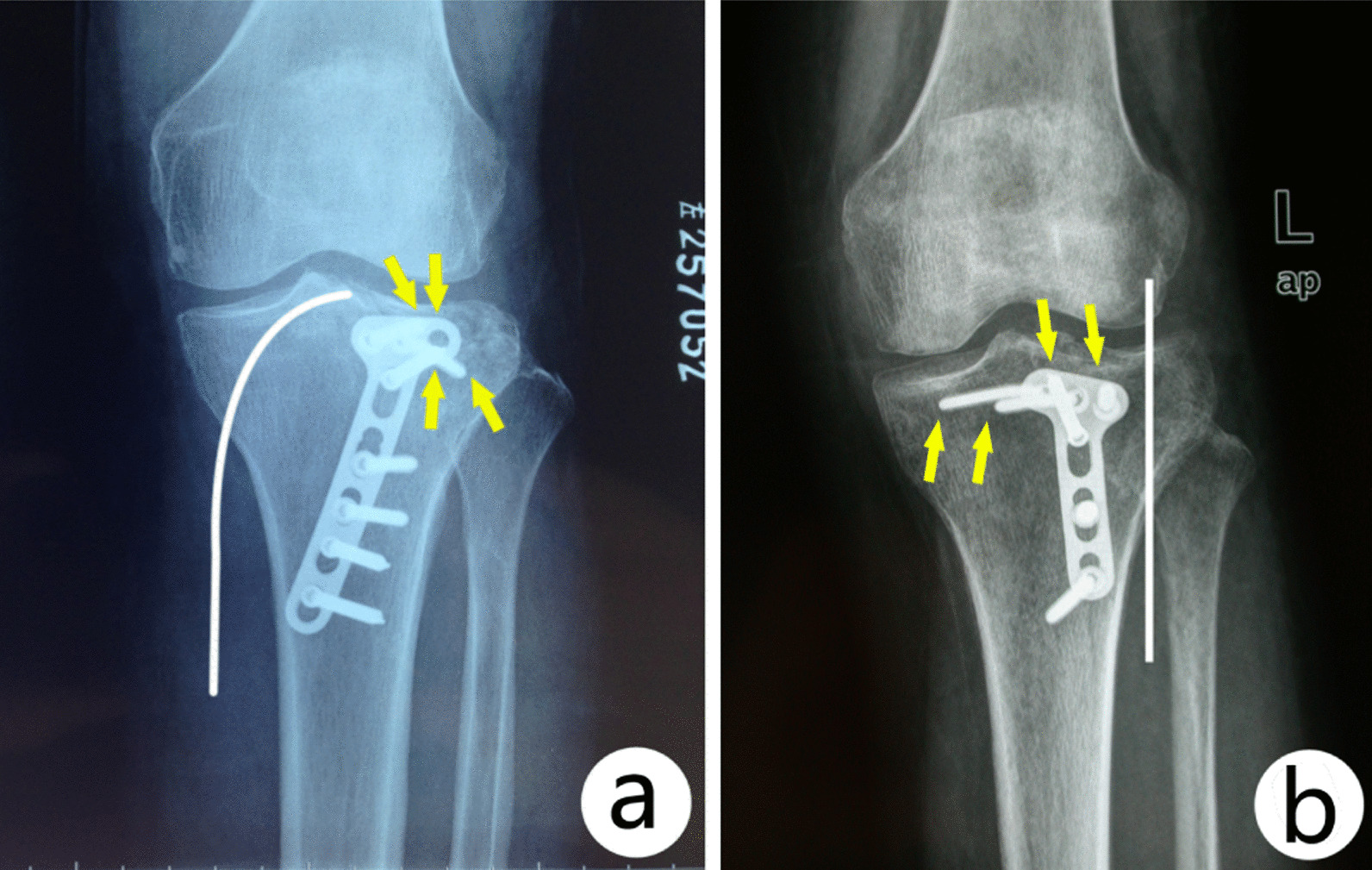
Comparison of plate placement and screw direction with each approaches: In a PL approach, the plate was placed vertically and the direction of screws toward middle or medial region (**b**). In a PM approach, the plate was placed obliquely with the screws direct toward posterolateral region (**a**). White line: skin incision; Yellow arrow: screw tip direction

For an isolated PL fracture, a straight T-shaped or L-shaped plate is often chosen and positioned vertically in a PL approach. Since the shielding by fibular head is over 50% area of PL tibial cortex, the plate is easily positioned toward medial side during surgery, and the direction of electric drill is easily toward the middle or medial side of tibial plateau when nailing. If a PM approach is chosen, the direction of electric drill is just from lateral to medial, which can more easily fix the PL fragment with the plate tilt positioned [27] (Fig. 5).

## Conclusions

In conclusion, although posterolateral approach seems more direct for exposure of posterolateral tibial plateau fracture, it has the risk of iatrogenic injury to several structures in deep dissection, including CPN, ATA, and PLC. Posteromedial approach through the medial board of medial head of gastrocnemius may be more safe, but it was hard for direct visualization of articular surface which limits it usage for only a few cases.

## Data Availability

The datasets used and/or analyzed during the current study are available from the corresponding author on reasonable request.
